# Retraction Note: Successful clavicle fracture surgery performed under selective supraclavicular nerve block using the new subclavian approach

**DOI:** 10.1186/s40981-020-00346-3

**Published:** 2020-06-03

**Authors:** Hironobu Ueshima, Hiroshi Otake

**Affiliations:** Department of Anesthesiology, Showa University Hospital, Hatanodai Shinagawa-ku, Tokyo, Japan

**Retraction Note: JA Clin Rep (2016) 2:34**

**https://doi.org/10.1186/s40981-016-0061-6**

The Editor-in-Chief has retracted this article [1]. Following an investigation, the Editor-in-Chief has been informed by the authors that the relevant patient files are missing; consequently, the Editor-in-Chief has concluded that the case report and its findings are unreliable as the information cannot be verified.

All authors agree to this retraction.
